# Maternal Psychological Problems Associated with Neonatal Intensive Care Admission

**DOI:** 10.1155/2009/591359

**Published:** 2009-07-27

**Authors:** Ziya Yurdakul, Ipek Akman, M. Kemal Kuşçu, Aytul Karabekiroglu, Gulsum Yaylalı, Figen Demir, Eren Özek

**Affiliations:** ^1^Department of Pediatrics-Neonatology, Marmara Medical School, Marmara University Hospital, Tophanelioğlu Cad. No:13/18, 34034 Istanbul, Turkey; ^2^Department of Psychiatry Consultation-Liason Unit, Marmara Medical School, Marmara University Hospital, Tophanelioğlu Cad. No:13/18, 34034 Istanbul, Turkey; ^3^Marmara Medical School, Marmara University Hospital, Tophanelioğlu Cad. No:13/18, 34034 Istanbul, Turkey; ^4^Department of Public Health, Marmara Medical School, Marmara University Hospital, Tophanelioğlu Cad. No:13/18, 34034 Istanbul, Turkey

## Abstract

*Background*. Mothers of infants admitted to a neonatal intensive care unit (NICU) are believed to have heightened distress. The purpose of this paper was to determine depression and anxiety symptoms and attachment style in NICU mothers. *Methods*. The NICU group consisted of mothers whose infants were admitted to the NICU and the control group consisted of mothers of healthy term infants. The psychosocial assessments were done at the first month. *Results*. The mean Edinburgh Postpartum Depression (EPDS) score of NICU mothers was significantly higher than that of the control group mothers (9.6 ± 5.6 versus 7.3 ± 4.9, *P* = .005). NICU mothers who had high EPDS (≥13) scores had significantly higher anxiety scores and insecure attachment style in comparison to the subgroup of NICU mothers who had low EPDS scores. *Conclusion*. Mothers of NICU babies had higher EPDS scores. Mothers who had higher EPDS scores had higher anxiety scores as well. These NICU mothers should receive appropriate counseling during the hospitalization of their babies.

## 1. Introduction

New motherhood involves many abrupt changes and is recognized a stressful life event [1, 2]. Recent reports indicate that 10%–15% of women suffer from postpartum depression (PPD), whereas approximately 10% develop an anxiety disorder after delivery [3, 4]. Risk factors for postpartum mood disorders include several sociodemographic and obstetric parameters. Although, the risk factors that predict PPD have been studied in detail in mothers of term healthy babies, there are limited studies about maternal psychological problems after the admission of the baby to NICU. Parents of infants admitted to an NICU are believed to experience the heightened distress compared to the parents of healthy infants. Carter et al. have reported that average level of anxiety and depressive symptoms in both the NICU and control parents was low, suggesting that for most parents the hospital experience was not associated with depression and anxiety symptoms. However they reported that a higher percentage of NICU parents had clinically relevant anxiety [5]. 

Insecure attachment style has been reported to be related to depression but its relationship to depression in mothers whose infants are admitted to NICU is largely unknown [6]. Our hypothesis is that secure adult attachment style could be buffering for mothers whose babies were admitted to NICU.

The purpose of this paper was to determine depression scores, anxiety scores, and the role of maternal attachment style in NICU mothers and to compare the results with those of mothers of healthy term babies.

## 2. Method

In this case-control study, mothers whose infants were admitted to the NICU at Marmara University Hospital were enrolled to the study as the study group. For each NICU baby-mother pair, a mother who delivered a healthy full-term baby on the same day was enrolled to the control group. Given the prevalence of postpartum depression of 10%, the minimum sample size should be 140 with 95% confidence interval and 5% standard deviation. But we chose to enroll 200 mother-infant pairs.

Among 100 NICU infants, 10 mothers refused to participate in the study. 2 mothers were excluded from the study because the infants died before 1 month of age. Among 100 control mothers, 9 mothers refused to participate in the study, 10 mothers did not come to the followup at first month after delivery as following. 

The flow diagram of case and control groups was shown in Table 1.

After written informed consent form had been obtained, two trained research assistants met each mother to conduct an assessment (i.e, Postpartum Assessment Instrument). The interview was either carried out in a separate room in the inpatient unit or in the Newborn Outpatient Clinic. The participants of the study were given a package of questionnaires composed of the Edinburgh Postpartum Depression Scale (EPDS), State-Trait Anxiety Inventory (STAI), Adult Attachment Scale (AAS), and Multidimensional Scale of Perceived Social Support (MSPSS) at the first month after delivery. Caregivers were instructed to fill out the questionnaires on their own time and reflect their own opinions and feelings without consulting anyone else. Caregivers who had difficulties understanding procedures and questionnaires were helped by research assistants via provision of further instructions to assure a valid assessment. The mothers in both control and study group had psychological testing at 1 month after delivery. Infants had a physical examination and nutritional assessment at 4 months of age. The psychological testing was not repeated at 4 months postpartum.

Mothers who were at high risk for PPD (EPDS ≥ 13) and needed clinical intervention were invited to the outpatient psychiatry clinic after the initial assessment for further clinical evaluation and followup. The study was approved by the Marmara University Ethical Committee, Istanbul, Turkey.

The assessments of NICU infant's health status and the mothers feeding preferences were also collected during this prospective study period for 4 months.

### 2.1. Postpartum Assessment Instrument

A semistructured interview was developed to assess the context of pregnancy and post-partum experiences. The instrument included questions about the demographic characteristics of mothers, medical adversities before the birth, quality of living circumstances, quality and quantity of social support network (resources and availability of supportive networks, family structure), problems and life events related to pregnancy, and life adversities before the childbirth (e.g., loss of significant others, accidents).

### 2.2. Edinburgh Postpartum Depression Scale (EPDS)

It is a 10-item scale developed to measure the depressive symptoms during post-partum period [7]. The scale focuses on specific depressive symptoms of postpartum period. Good postpartum sensitivity and specificity have been reported for the scale in the UK [7, 8]. The Turkish validation is done by Engindeniz et al. [9].

### 2.3. Adult Attachment Scale (AAS)

AAS is a Likert-type self-report scale developed by Collins and Feeney based on Hazan and Shaver's Attachment Style Measure [10, 11]. It assesses three adult attachment styles, namely, secure, avoidant, and ambivalent styles. The Adult Attachment Scale was translated into Turkish and validated by Alp [12]. It assesses three adult attachment styles, namely, secure, avoidant, and ambivalent styles. The avoidant and ambivalent styles were grouped as insecure attachment style in this study.

### 2.4. State-Trait Anxiety Inventory (STAI)

The STAI is a 40-item questionnaire measuring state (20 items) and trait (20 items) anxiety. The STAI assesses how respondents feel right now (state) and how respondents generally feel (trait) on a 4-point Likert Scale, indicating experience of a significant amount of anxiety symptom. The total score for trait and state anxiety ranges from 20 to 80. The STAI has good internal consistency and test-retest reliability [13, 14].

### 2.5. Multidimensional Scale of Perceived Social Support (MSPSS)

The MSPSS is a self-report measure of perceived social support composed of 12 items, with four items comprising each of three sources of social support (family, friends and significant others) [15]. The MSPSS was translated to Turkish, and its validity and reliability was provided by Eker and Arkar [16].

## 3. Statistical Analyses

Statistical analyses were done by SPSS for Windows, version 11.5 (SPSS Inc., Chicago, Illinois, USA). Data were presented as means standard deviations and percents. Student's *t*-test, Mann-Whitney *U*-test, Chi-square and Fisher's exact test were used to identify differences between two groups. Nonparametric tests were used when data were not normally distributed. Spearmans rho correlations were computed to evaluate the associations of EPDS total scores with STAI, MNPSS scales that are used in this study. The level of significance was taken as *P* < .05.

## 4. Results

There were no significant differences in maternal age, working status, education level, parity, between the NICU and control groups. We did logistic regression as a multivariate analysis to find out confounding factors for EPDS score of mothers whose babies were admitted to NICU trough maternal age, working status, education level, parity, duration of hospital stay, birth weight, gestational age, sex of babies and maternal health problems during pregnancies except for socioeconomic status of mothers. One of limitations of this study was that we did not take into account the socioeconomic status of mothers.

There were significantly less babies who were exclusively breastfed at 4 months of age in the NICU group (Table 2). The mean birth weights of the study and control group infants were 3390 ± 510 and 2570 ± 990 (*P* < .0001), respectively, and all the control group infants were full term. Of the NICU infants, 49% (*n*: 43) was preterm and their mean birth weight was 1958 ± 696 g, their mean gestational age was 32.6 ± 2.7 week, 51% (*n*: 45) was born at term and their mean birth weight was 3142 ± 578 g, their mean gestational age was 38.7 ± 1.2 week. The difference between the mean birth weight of the study and control infants is due to the presence of premature babies.

The admission diagnosis to the NICU was respiratory distress syndromes (23%), suspected sepsis (10%), hyperbilirubinemia (23%), surgical conditions (15%) and hypoxic-ischemic encephalopathy (5%) and other conditions like hypoglycemia, feeding difficulties, and so forth (24%).

The number of mothers with high depressive scores (EPDS ≥ 13) and the mean EPDS scores was significantly higher in the NICU mothers compared to the control mothers (29.5% versus 13.6%, *P* = .012, and 9.6 ± 5.6 versus 7.3 ± 4.9, *P* = .005). However, state-trait anxiety scores and attachment styles were not different between the NICU and control mothers (Table 3). 

The state and trait anxiety scores were correlated with EPDS scores in the NICU mothers (*r* = 0.37/*P* = .003, *r* = 0.32/*P* = 0.003, resp.). 

There was also no significant difference between the mean EPDS scores of NICU mothers whose babies were born at term or before 37 weeks of gestation (9.6 ± 5.3 versus 9.6 ± 5.9, *P* = .97).

We divided the NICU mothers into the high EPDS subgroup (EPDS ≥ 13) and low EPDS subgroup (EPDS < 13). The subgroup with high EPDS scores in the NICU mothers had significantly higher anxiety scores and insecure attachment style than the low EPDS subgroup in the NICU mothers (*P* < .05). Duration of NICU stay was also significantly higher in the high EPDS subgroup compared to the low EPDS subgroup in the NICU mothers. But, no statistical differences between these high EPDS and the low EPDS subgroups in the NICU group regarding educational levels and parity were found (Table 4).

We also did not find any statistical differences between the high and low EPDS subgroups in the control mothers regarding anxiety scores, MSPSS, maternal age, educational levels, parity and type of delivery except for the higher insecure attachment style in the high EPDS subgroup of control mothers (*P* = .001) (Table 5).

## 5. Discussion

In this study, the depression, anxiety, attachment, and social support scores of a group of NICU mothers were compared to the mothers of healthy term infants. The mean EPDS score of the NICU mothers was significantly higher than that of the control mothers while state-trait anxiety scores, attachment styles, and MSPSS scores were not different between the NICU and control mothers. 

In the literature the prevalence of PPD has been estimated at 10% to 15%, and prevalence of various anxiety disorders among pregnant women has been estimated to be 10% [2, 4, 17, 18]. In a study, 22% of NICU mothers had possible depression based on the EPDS, and this was not statistically different than the risk of depression of mothers of healthy infants [5]. However in our study 29.5% of NICU mothers had possible depression based on the EPDS, and this was significantly higher than the risk of depression of the control group. This may be due to having a sick infant, the stress of the NICU environment, the physical and emotional isolation of the mothers from the baby. In this study the high EPDS scores do not appear to be related to woman's educational levels, the sex of infant, the mode of delivery but the duration of hospital stay was associated with the high EPDS scores. The birth and subsequent hospitalization of a premature infant evoke considerable psychological distress in the mothers. In particular, lack of social support, previous history of depression, marital conflict, and stressful life events have been found to significantly increase risk of postpartum depression [4].

Although the nature of relationships between having an infant in the NICU and anxiety in parents is uncertain, Carter et al. showed higher anxiety in the NICU parents compared to the healthy infant parents [5]. We also found higher anxiety scores and insecure attachment style in the NICU mothers compared to control mothers but it was not statistically significant. Neonatal intensive care experience is a significant factor of discontinuity for mother-infant dyadic relationship. So far, the studies which explore the maternal attachment style and its role in neonatal intensive care units are limited [19–24]. Our hypothesis is that secure adult attachment style could be buffering for mothers whose babies were admitted to NICU. In our previous study, we described that the mean EPDS score of mothers who live in extended families is found to be significantly lower than the mothers who live in nuclear families and there were positive correlations with EPDS scores and insecure attachment [12, 25, 26]. In this study, the subgroup of NICU mothers with high EPDS scores had significantly higher anxiety scores and insecure attachment style than the low EPDS subgroup of NICU mothers (*P* < .05). 

Therefore it is very important to detect depressed mothers by NICU professionals for the better mother-child interaction. Given the high EPDS scores in this study, it is recommended that mothers of NICU infants should be routinely screened for postpartum depression. The findings also have implications for nursing, medical, and other health care professionals working in the NICU like other studies [27, 28]. 

Estimates of between 28% and 70% of mothers of premature infants have been reported as having clinically significant degrees of psychological distress [26]. On the other hand, the hypothesis that mothers with smaller and sicker infants would be at greater risk for depressive symptoms was not conclusively supported [29]. 

The prevalence and clinical presentation of anxiety disorders during the postpartum period have received little research attention. Anxiety disorders are common during the perinatal period, with reported rates of obsessive-compulsive disorder and generalized anxiety disorder being higher in postpartum women than in the general population [30]. Depression and/or anxiety were prevalent in 16.5% of postpartum women versus 29.2% of pregnant women [31]. Patients with “postpartum depression” usually had at least one other (comorbid) disorder, and 27% had two or more. After delivery the commonest themes were the pathological fear of cot death and fear of the criticism of mothering skills which was a clue to a disordered mother-infant relationship [32]. Certain mothers are at particularly high risk for anxiety in the immediate postpartum period: those who have experienced preterm birth or other perinatal complications, as well as those lacking a satisfactory marital relationship or other forms of social support [33]. 

In this study exclusive breastfeeding was also significantly lower in NICU group compared to the control group. Maternal depression has been recognized in other studies as influencing maternal feeding attitudes [34, 35] and also the duration of breastfeeding [36]. 

Adequacy of milk supply and perinatal medical condition of the infant was a key factor for successful breastfeeding of preterm infants. Akerstrom and Norman have reported that 6 months after discharge from hospital 89% of term infants and 47% of preterm infants were breastfeeding exclusively or in part [37]. According to our results high maternal EPDS score may affect breastfeeding in the NICU but lower breastfeeding rate may be also due to other factors like medical problems of the baby. In our unit we recommend breastfeeding to all mothers including those who bear a preterm infant. When the baby's clinical status is not suitable for breastfeeding, we use pumped breast milk and give it to the baby by orogastric tube. Sometimes mothers do not pump their milk regularly leading to decreased milk supply. 

In conclusion, in this study NICU admission of baby was found to be associated with the higher EPDS score of the mother and these mothers with the higher EPDS scores had higher anxiety scores and insecure attachment styles. NICU professionals should be more careful about depressive symptoms of NICU mothers and should provide counseling when it is necessary. Further studies on bigger samples are required to test the impact of stress of the NICU on the mothers.

## Figures and Tables

**Table 1:** 

NICU group	Control group
100	100
↓	↓
10 refused	9 refused
2 infant died	10 did not come to followup in first month
↓	↓
88 mother-infant pairs were assessed	81 mother-infant pairs were assessed

**Table 2:** Demographic characteristics of the NICU and control groups.

	NICU group *n*: 88	Control group *n*: 81	*P*
Mother age (mean ± SD) years	30.08 ± 5	29.9 ± 5	.90

Type of delivery:			
NVD (*n*)	22	47	<.**001**
Caesarean (*n*)	66	34	

Parity:			
Multipar (*n*)	37	40	.33
Primipar (*n*)	51	41	

Gender of the baby:			
Male (*n*)	45	40	.82
Female (*n*)	43	41	

Education of mother (*n*):			
<8 years	28	20	.30
≥8 years	60	61	

Working status:			
Working (*n*)	44	45	.82
Housewife (*n*)	44	36	

Exclusive breastfeeding (*n*)	43	68	<.**001**
Breastfeeding and/or formula (*n*)	45	13	
(First month)			

Exclusive breastfeeding (*n*)	36	56	<.**001**
Breastfeeding and/or formula (*n*)	52	25	
(4th month)			

**Table 3:** Psychological adjustment of NICU and control mothers.

	NICU mothers *n*: 88	Control mothers *n*: 81	*P*
EPDS (mean ± SD)	9.6 ± 5.6	7.3 ± 4.9	.**005**

Anxiety inventory:			
State (mean ± SD)	46.4 ± 12.7	43.2 ± 12.1	.10
Trait (mean ± SD)	39.8 ± 9.2	38.9 ± 9.2	.50

Adult attachment scale:			
Secure (*n*)	57	57	.36
Insecure (*n*)	31	24	

EPDS scores (EPDS ≥ 13) (*n*)	26	11	.**012**

MSPSS (mean ± SD)	71.24 ± 14.6	71 ± 12.8	.91

**Table 4:** Psychological adjustment and demographic characteristics of NICU subgroups mothers according to the EPDS scores.

	High EPDS subgroup (≥13) *n*: 26	Low EPDS subgroup (<13) *n*: 62	*P*
Gestational age:			
Preterm (<37 wk) (*n*)	15	28	.28
Term (≥37 wk) (*n*)	11	34	

Infant hospital stay: days			
Median (min-max)	12 (1–75)	7 (1–65)	**.022**

Maternal age (mean ± SD) years	30.8 ± 5.3	29.7 ± 4.8	.40

Parity:			
Multipar (*n*)	15	22	.054
Primipar (*n*)	11	40	

Type of delivery:			
NVD (*n*)	5	17	.41
Caesarean (*n*)	21	45	

Education of mother:			
<8 years (*n*)	11	17	.17
≥8 years (*n*)	15	45	

Anxiety inventory: (mean ± SD)			
State	52.5 ± 12.8	43.7 ± 11.8	**.003**
Trait	43.2 ± 11.6	38.3 ± 7.5	**.024**

Adult attachment scale:			
Secure (*n*)	9	48	**.001**
Insecure (*n*)	17	14	

MSPSS (mean ± SD)	68.4 ± 12.6	72.6 ± 15.5	.22

**Table 5:** Psychological adjustment and demographic characteristics of control subgroups according to the EPDS scores.

	High EPDS subgroup (≥13) (*n*: 11)	Low EPDS subgroup (<13) (*n*: 70)	*P*
Anxiety inventory: (median(min-max))			
State	42.5 (25–75)	43 (22–75)	.47
Trait	34.5 (27–61)	47.5 (20–61)	.17

Adult attachment scale:			
Secure (*n*)	3	54	**.001**
Insecure (*n*)	8	15	

MSPSS (median(min-max))	72.5 (45–84)	75 (12–84)	.63
Maternal age (med(min-max)) years	28 (19–39)	30 (21–41)	.31

Education of mother:			
<8 years (*n*)	3	17	.83
≥8 years (*n*)	8	53	

Parity:			
Multipar (*n*)	5	36	.71
Primipar (*n*)	6	34	

Type of delivery:			
NVD (*n*)	5	42	.29
Caesarean (*n*)	6	28	
